# The epidemiology of suicide and attempted suicide in Dutch general practice 1983–2003

**DOI:** 10.1186/1471-2296-6-45

**Published:** 2005-11-04

**Authors:** Richard L Marquet, Aad IM Bartelds, Ad JFM Kerkhof, François G Schellevis, Jouke van der Zee

**Affiliations:** 1 Netherlands Institute of Health Services Research (NIVEL), PO Box 1568, 3500 BN, Utrecht, The Netherlands; 2 Department of Clinical Psychology, Free University Amsterdam, van der Boechorststraat 1, 1081 BT Amsterdam, The Netherlands; and Research Institute Psychology and Health, Utrecht, The Netherlands

## Abstract

**Background:**

Many patients attempting or committing suicide consult their general practitioner (GP) in the preceding period, indicating that GPs might play an important role in prevention. The aim of the present study was to analyse the epidemiology of suicidal behaviour in Dutch General Practice in order to find possible clues for prevention.

**Method:**

Description of trends in suicide and suicide attempts occurring from 1983–2003 in the Dutch General Practice Sentinel Network, representing 1% of the Dutch population. The data were analysed with regard to: 1) suicidal behaviour trends and their association with household situation; 2) presence of depression, treatment of depression and referral rate by GPs; 3) contact with GP before suicide or suicide attempt and discussion of suicidal ideation.

**Results:**

Between 1983 and 2003 the annual number of suicide and suicide attempts decreased by 50%. Sixty percent of the patients who committed or attempted suicide were diagnosed as depressed, of whom 91% were treated by their GP with an antidepressant. Living alone was a risk factor for suicide (odds ratio 1.99; 95% CI 1.50 to 2.64), whereas living in a household of 3 or more persons was a relative risk for a suicide attempt (odds ratio 1.81; 95% CI 1.34 to 2.46). Referral to a psychiatrist or other mental health professionals occurred in 65% of the cases. GPs recalled having discussed suicidal ideation in only 7% of the cases, and in retrospect estimated that they had foreseen suicide or suicide attempts in 31% and 22% of the cases, respectively, if there had been contact in the preceding month.

**Conclusion:**

With regard to the prescription of antidepressants and referral of suicidal patients to a psychiatrist, Dutch GPs fulfil their role as gatekeeper satisfactorily. However, since few patients discuss their suicidal ideation with their GP, there is room for improvement. GPs should take the lead to make this subject debatable. It may improve early recognition of depressed patients at risk and accelerate their referral to mental health professionals.

## Background

As in most Western countries, suicide and suicide attempts are major public health problems in the Netherlands [1]. Suicide is the 14^th ^leading cause of death and the fifth leading cause of years of potential life lost [[2], Statistics Netherlands]. Attempted suicide, being an important risk factor for suicide, is estimated to be as high as 130/100,000 which represents a significant burden to the individuals affected, their families and the health services [3]. Many of these patients consult their general practitioner (GP) in the period preceding their suicide or suicide attempt, indicating that GPs might play an important role in prevention [4]. However, knowing that a Dutch GP may lose a patient to suicide only once every 4–5 years, it is clear that early recognition of suicidal patients is difficult [5]. Still, GPs may have ways to prevent suicide and suicide attempts: 1) by addressing patients' emotional problems, 2) screening for depression, 3) probing for suicide ideation and 4) making proper referrals to mental health professionals [6,7]. Our question was: to what extent do Dutch GPs fulfil these requirements? Ever since 1979, suicide and suicide attempts occurring in Dutch general practice are registered by GPs participating in the Dutch Sentinel Network [5]. In order to find clues for early recognition of suicide-endangered patients we analysed relevant data from the Network over the period 1983–2003.

## Methods

The data were derived from the Dutch Sentinel Practice Network, which has been operational since 1970; it constitutes a sample of about 65 GPs, covers about 1% of the Dutch population, and is representative of the total population (16.3 million in 2003), with regard to age, sex, geographical distribution, and level of urbanisation. Over 95% of non-institutionalised citizens in the Netherlands are registered with a GP. The Dutch Sentinel Network structure takes account of the geographical distribution of the population and its spread over areas with different degrees of urbanisation. A census is held every two years among the practice populations involved, in order to determine the size and composition of the population to which the gathered data is to be related. The Dutch Sentinel Network has been participating in many national [5,8,9] and international projects. The oldest international project is the European Influenza Surveillance Scheme [EISS; [10]]. Using specific forms, GPs participating in the network report weekly to our Institute on the incidence of various diseases, types of diagnosis and events, including requests for euthanasia, and suicide and suicide attempts. For every case of suicide or suicide attempt reported anonymously by the sentinel GPs, additional data are provided with regard to: 1) age and sex, 2) previous attempts, 3) method and place, 4) number of persons in the household, 5) previous contact with the GP, 6) nature of this contact, 7) presence of depression (defined according to guidelines by the Dutch GP Association [NHG, [11]], and its treatment, 8) referral to mental health professionals, and 9) whether the GP had foreseen the suicide or suicide attempt.

## Results

### Suicides and suicide attempts: numbers, age distribution and relationship with household

From 1983–2003 a total of 248 suicides (169 men and 79 women) and 1,231 suicide attempts (409 men and 822 woman) were registered. As far as GPs were informed, most suicides (total 75%; 80% for men, 65% for women) were committed at first attempt. Likewise, 67% of male non-fatal suicidal behaviour and 59% of female non-fatal behaviour were considered to be first attempts.

**Figure 1:**
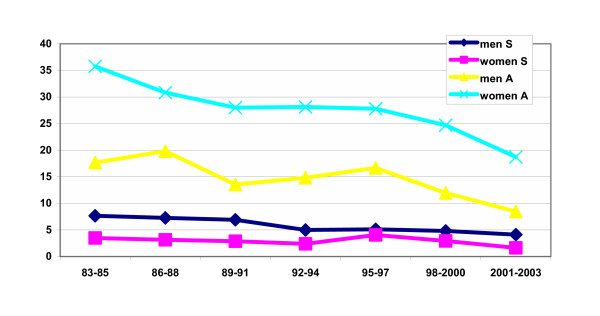
Suicide (S) and attempted suicide (A) in Dutch general practice 1983–2003; 3-year averages/100,000 inhabitants.

In figure 1 the 3-year averages of the number of suicides and suicide attempts per 100,000 inhabitants are depicted. It can be observed that both rates gradually decreased over time. Compared to the 1983–1985 period the average number of suicides and suicide attempts in the 2001–2003 period was almost 50% lower, both for men and women. A similar trend has been identified nationally, indicating that the sample from the sentinel network is representative [2]. The total number of suicides decreased from 11.2 to 5.7/100,000; the number of suicide attempts decreased from 53.5 to 27.0/100,000.

**Figure 2:**
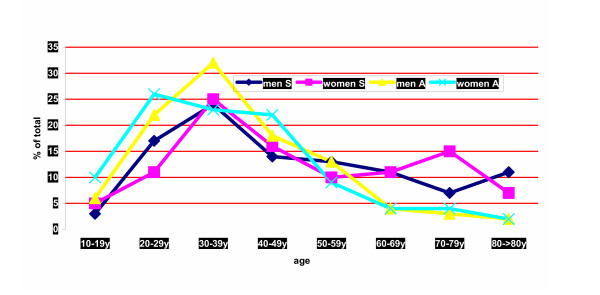
Age distribution of suicides (S) and attempted suicides (A) of men an women 1983–2003; % of total.

**Figure 3:**
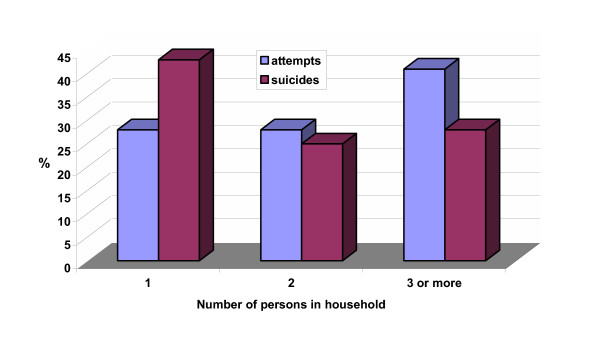
Suicides and suicide attempts; relationship with number of persons in household

The age distribution of suicides and suicide attempts is given in figure 2. About 50% of all suicides and 70% of all suicide attempts were committed between the age of 20 and 50 years. Prominent peaks occurred in the age group of 30–39 years, except for suicide attempts in women, which reached their highest proportion between 20 and 29 years. It is noteworthy that in persons older than 60 years the proportion of attempted suicides decreased, whereas suicide in elderly men and women did not. Figure 3 shows the percentage of persons who committed suicide or attempted suicide in the context of their domestic situation. Suicides were more common in persons living alone (odds ratio 1.99; 95% CI 1.50 to 2.64), whereas suicide attempts occurred more frequently in households consisting of 3 or more persons (odds ratio 1.81; 95% CI 1.34 to 2.46).

### Depression

**Figure 4:**
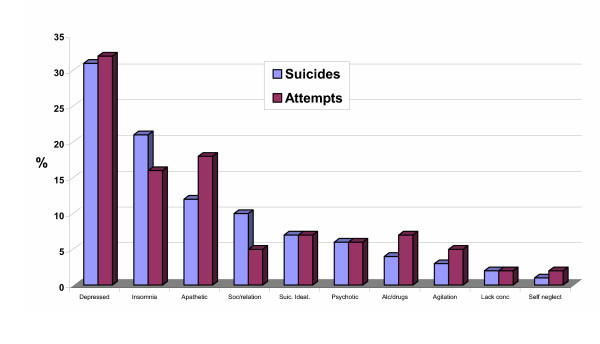
Characteristics and symptoms of suicidal patients suffering from depression.

More than 50% of the patients (60% of total; 52% men, 68% women) who committed or attempted suicide were diagnosed by their GP as being depressed. On average, the prevalence of depression was about 8% higher in patients who committed suicide than in those who attempted suicide. The characteristics and symptoms of suicidal patients suffering from depression are presented in figure 4. Most of the characteristics were similar for those who died by suicide and for those who attempted suicide. Nearly all depressed patients (91%) were treated with an antidepressant. From 1983 to 1993 the first drug of choice prescribed by the GP was a tri-cyclic antidepressant (TCA); selective serotonin reuptake inhibitors (SSRIs) became increasingly popular thereafter and comprised 83% of the prescriptions for depression in 2003. An important finding was that in only 7% of cases GPs recalled to have discussed suicidal ideation with depressed patients who committed or attempted suicide.

### Referral of patients; did the GP foresee suicide or the suicide attempt?

Sixty-five percent of the patients who ultimately committed suicide and 63% of those who attempted suicide had been referred by their GP to a psychiatrist or an institution for ambulatory mental health care. In the Netherlands, mental health care professionals at these institutions include psychiatrists, psychologists and social workers. The referral frequency steadily increased over time, from 70% in 1983 to 84% in 2003.

Fifty-three percent of the patients who committed suicide and 54% of those who attempted suicide had contacted their GP in the 30-day period preceding the event. In the case of contact GPs mentioned retrospectively that suicide was foreseen in 31% of the cases; when there had been no contact suicide was foreseen in 7% of the cases. For suicide attempts these percentages were 22% and 3%, respectively.

## Discussion

A few characteristics of a person at risk of suicide or a suicide attempt do emerge from the present study. Somewhat overstated, the typical profile of a person at risk of suicide in the Netherlands is a young or elderly depressed patient, frequently living alone, most likely to be a man, treated by the GP with a modern antidepressant and rarely speaking of suicidal ideation. Living alone as a risk factor has been cited in many epidemiological studies on suicide. (12, 13, 14). Strong associations between suicide/suicide ideation and different aspects of loneliness, either subjective (feeling lonely) or objective (living alone and being without friends) have been observed (13). The profile of a person at risk for a suicidal attempt is almost the same, except that this depressed patient is more frequently a younger woman not living alone. Most of these characteristics are certainly not exclusively Dutch, they have been cited before by several investigators from different countries [12-19].

Typical for the Dutch situation is that for the first time a more precise estimate can be made about the incidence of attempted suicides in the Netherlands. Earlier estimates were based on a four-year inventory (1989–1991) among general hospitals, psychiatric hospitals and GPs in a defined area around the city of Leiden (3). From these data an incidence of 143/100,000 was calculated. Our results indicate that the mean yearly incidence of attempted suicides reported to GPs in the period 1983–2003 is 41/100,000. In the Leiden study it was observed that about 40% of all suicide attempts was reported by GPs. If this percentage also applies for the whole country it can be calculated that the incidence of attempted suicides in the Netherlands is lower than estimated in the Leiden study, namely about 100/100,000, an incidence almost similar to that recently calculated for France [20]. Interestingly, the number of attempted suicides reported by GPs in the period 2001–2003 was 50% less than reported in 1983–1985, both for men and women. This coincides with the increased prescription of SSRIs, however there is no proof that this coincidence, which also applies for the reduced number of suicides, has causal aspects. We do not know whether this decreasing trend fully represents the decline in the incidence of (attempted) suicides, or is also due to a shift from GP to secondary mental health care and psychiatric hospitals. This factor of "missed patients" might also explain why the official trend line for suicides calculated by Statistics Netherlands lies below the trend line calculated from the GP data [[2], Statistics Netherlands].

Depression is the most common psychiatric disorder in patients who attempt or commit suicide [21,22]. The role of the GP in the treatment of depression therefore is of considerable clinical importance. We found that 60% of patients who attempted or committed suicide were diagnosed by their GP as being depressed. This is fairly consistent with percentages cited by others [20]. In the year 2000 the Dutch Sentinel Network registered 440/100,000 new cases of depression [9]. If we relate this number to our data, while realising that being depressed is not the same as being diagnosed as depressed, it can be calculated that about 1% of depressed patients commit suicide and about 5% of depressed patients will attempt suicide. These figures are comparable with those of Khan et al. in the USA who calculated that 0.8% of patients participating in antidepressant trials committed suicide and 2.9% attempted suicide [23]. How does the Dutch GP cope with depressed patients who ultimately commit or attempt suicide, considering that a percentage of 6% is still tantamount to a needle in a haystack? From our survey the following picture emerges: Dutch GPs prescribe antidepressants fairly readily; they have switched almost unanimously to prescribing SSRIs, and refer depressed patients readily to a mental health professional. This sounds like an ideal situation, which is in keeping with the gatekeeper's role of GPs in the Netherlands [24]. With regard to serious depression this policy means that the GP provides the basic medication and leaves the in- depth treatment and responsibility to a psychiatrist and allied mental health caregivers. Still, there is room for improvement. The Dutch GP is either not a great communicator with regard to discussing suicidal ideation, or simply lacks the time to address this emotive subject: GPs recalled to have discussed suicidal ideation with only 7% of the depressed patients that ultimately committed or attempted suicide, which is a low percentage as far as risk assessment is concerned. However, it should be noted that this percentage is only an estimate. It is based on GP's recollection of having discussed suicidal ideation after the patient had attempted or committed suicide. Similar scepticism is warranted on the retrospective estimate whether the GP might have foreseen the suicide or suicide attempt. The relatively high percentages of 'foreseeing' in our analysis if there had been contact in the month preceding the suicide or suicide attempt (31% and 22%, respectively), are also based on GPs' recollection and therefore liable to bias.

## Conclusion

With regard to prescribing antidepressants and referring depressed patients to a psychiatrist, Dutch GPs fulfil their role as gatekeeper satisfactorily. However, since few patients discuss their suicidal ideation with their GP, there is room for improvement. Our results suggest that GPs should ask more persistently for suicidal ideation in depressed patients, especially in those living alone. Making this issue debatable may enhance early recognition of high-risk patients and accelerate their referral to mental health professionals.

## Competing interests

The author(s) declare that they have no competing interests.

## Authors' contributions

RLM and AIMB performed the data analysis. AIMB also developed the study concept and headed its coordination. JvdZ and FGS participated in the design and coordination of the study. AJFMK contributed to the design and draft of the manuscript.

All authors read and approved the final manuscript.

## Pre-publication history

The pre-publication history for this paper can be accessed here:


